# Self-Reported Low Self-Esteem. Intervention and Follow-Up in a Clinical Setting

**DOI:** 10.1100/tsw.2007.88

**Published:** 2007-02-09

**Authors:** Søren Ventegodt, Suzett Thegler, Tove Andreasen, Flemming Struve, Lars Enevoldsen, Laila Bassaine, Margrethe Torp, Joav Merrick

**Affiliations:** ^1^ Quality of Life Research Center, Teglgårdstræde 4-8, DK-1452 Copenhagen K, Denmark; ^2^ Research Clinic for Holistic Medicine, Copenhagen, Denmark; ^3^ Nordic School of Holistic Medicine, Copenhagen, Denmark; ^4^ Scandinavian Foundation for Holistic Medicine, Sandvika, Norway; ^5^ Interuniversity College, Graz, Austria; ^6^ Zusman Child Development Center, Soroka University Medical Center, Ben Gurion University of the Negev, Beer-Sheva, Israel; ^7^ National Institute of Child Health and Human Development, Jerusalem, Israel; ^8^ Office of the Medical Director, Division for Mental Retardation, Ministry of Social Affairs, Jerusalem, Israel

**Keywords:** self-esteem, psychodynamic therapy, bodywork, holistic health, human development, Denmark, Short-term psychodynamic psychotherapy (STPP)

## Abstract

At the Research Clinic for Holistic Medicine in Copenhagen, 43 patients who presented with low or very low self-esteem were treated with psychodynamic short-term therapy complemented with bodywork. They received an average of 20 sessions at a cost of 1,600 EURO. The bodywork helped the patients to confront old emotional pain from childhood trauma repressed to the body-mind. Results showed that 60.5% recovered from low selfesteem (95% CI: 44.41–75.02%). Calculated from this, we have NNT = 1.33–2.25. Almost all aspects of life improved at the same time (p < 0.01): physical health, mental health, quality of life, and ability to function in a number of important areas (partner, friends, sexually, and socially). This indicated that we had successfully induced existential healing (Antonovsky salutogenesis). The strategy of improving self-esteem can be the key to a new life for patients presenting with low quality of life, poor health (physical and/omental), and poor ability to function. The patients were strongly motivated and willing to endure strong emotional pain provoked by the therapy. The rate of recovery is comparable to the most successful interventions with psychological and psychiatric treatment. Clinical holistic treatment has many advantages: efficiency, low cost, lack of negative side effects, lasting results, lack of use of psychopharmacological drugs (often with side effects), and an important preventive dimension.

## INTRODUCTION

The most fundamental problem of human existence seems to be how to love oneself[1,2]. The reason for this is our triple nature[3,4]: having body, mind, and soul, each carrying its own representations of self: the Id, the Ego and – Me! So who am I? When a patient starts to become whole, he subjectively recovers his sense of coherence[5,6]. Looked on from the outside, he becomes more alive, more real, and solid. But much more than that is happening: the patient is gaining health, quality of life, and ability on all areas of life. Sexual and social ability are often radically improved with the ability to love and work also rehabilitated. This process of gaining existential health was called *salutogenesis* by Aaron Antonovsky (1923–1994)[5,6]. Taking the patient into such a process of existential healing might be the medical strategy for the new millennium[7,8].

## METHODS

Psychodynamic short-term therapy[9,10,11] combined with "spiritual" mindfulness[12,13] and bodywork[14,15,16] was used in this study in order to work with all aspects of body, mind, and spirit at the same time, as conversational therapy is often mainly mind-work. From 1990 to 2004, we analyzed how more than 2000 life factors affected quality of life and health in order to conclude that philosophy of life was the single most important causal factor[17], which we afterwards have used in therapy. From 1997 to 2005, we treated more than 500 patients using this new combined method, which we have called clinical holistic medicine[18,19,20]. We have recently been able to demonstrate that this intervention is safe and efficient with patients suffering from physical, mental, and sexual problems with the effect of therapy lasting for more than a year[21]. The clinic has an open-door policy and the patients all come "from the street" having read our books[18,19,20,22,23] or, most commonly, by recommendation from other patients (word by mouth). They entered this study if they rated 4 or 5 on the five-point Likert Scale for quality of life (QOL5)[24]: How do you feel about yourself at the moment? 1, "Very good"; 2, "Good"; 3, "Neither good nor bad"; 4, "Bad"; 5, "Very bad". Eight therapists performed the therapy under supervision (by SV). There were four major common themes in the therapy: sex and the body, consciousness and mind, love and spirit, and using your own talents and true self to be of real value to others[18,19,20].

The patients were measured with a five-item quality of life and health questionnaire (QOL5) (five questions on self-assessed physical health, mental health, relation to self, relation to partner, and relation to friends[24]), a one-item questionnaire of self-assessed quality of life (QOL1)[24], and four questions on self-rated ability to love, self-rated ability to function sexually, self-rated social ability, and self-rated working ability (ability to sustain a full-time job) (together, QOL10). These questionnaires were administered before entering the study, after the treatment, and after 1 year[20,21].

## RESULTS

### How Many Recovered Their Self-Esteem?

Out of 43 patients who entered the study (see Table 1), only three continued to feel bad about themselves after an average of 20 sessions. Fourteen patients did not complete therapy or failed to fill in the follow-up questionnaire. The therapy confronted the patients with their many repressed and often painful emotions from childhood of anger, guilt, shame, hopelessness, despair, and anxiety. Two patients had severe existential crises lasting for a few days, but soon recovered, and no patient was harmed from the intervention or had severe side effects. Not all patients were sufficiently motivated to confront the painful emotions from the past, which made them drop out. Success rate of treatment: 26/43 = 60.47% (95% CI: 44.41–75.02%)[25]. The Number Needed to Treat (NNT) of clinical holistic medicine with patients with low self-esteem is therefore NNT = 1.33–2.25. As we have treated more than 500 patients with no patient harmed, we estimate the Number Needed to Harm (NNH) to be >500.

**TABLE 1**
**Clinical Holistic Medicine Cure of Patients from Self-Assessed Self-Esteem**

| | Before Treatment | After Treatment |
|---|---|---|
| Low or very low self-esteem | 43 | 3 |
| Very high, high, or intermediate self-esteem | 0 | 26 (17 high or very high, 9 intermediate); 26/43 = 60.47% (95% CI*: 44.41–75.02%)[25] |
| Nonresponders or dropouts | — | 14 |
| Low or very low self-esteem, nonresponder, or dropout | 43 | 17 |

* Confidence interval.

## What Happened to the Responders?

Most interestingly, the patients who responded to the holistic existential therapy and improved their self-esteem (relationship with self) also improved all other areas of life: quality of life (both self-assessed with QOL1[20,24] and measured by the validated questionnaire QOL5[20,24]), self-evaluated physical and mental health, and self-evaluated ability to function. All these improvements were about one step up the five-point Likert scale, making them both statistically and clinically highly significant (see Tables 2, 3, 4, and 5).

We found (Tables 4 and 5) that the 26 patients who recovered their self-esteem in the therapy also improved their relationships in general (with self, partner, and friends), their self-evaluated ability to function in general (love, sex, and social ability), and their quality of life as measured with QOL5. When health, quality of life, and ability were combined (in the measure called QOL10 that takes the average of these three domains), it was clear that the patients had healed their whole life (as measured by QOL10[21]), not only their self-esteem.

Tables 2 and 3 show that the 26 patients who recovered their self-esteem also improved their self-evaluated physical and mental health, relationship with friends and partner, ability to love, ability to function sexually, social ability, and self-assessed quality of life (QOL1)[24]. Please notice that the results are both statistically and clinically highly significant (self-assessed physical health $p < 0.05$, working ability is not improved significantly, all other results $p < 0.01$).

## DISCUSSION

Although research has stressed the connection between health and self-esteem with development of self-esteem often suggested as one of the most important ways to prevent illness, improve health, and fortune, it has been difficult to understand, conceptualize, measure, or improve self-esteem[26,27,28,29,30,31]. Even though self-esteem is strongly related to quality of life, health, and ability, the connection between them still remains quite obscure[26,28,29,30,31,32].

It seems that in order to change a person’s self-esteem, the most fundamental dimensions of existence must be analyzed and developed, and such a development of the person’s innermost layer seems to be a true transformation of personality. We have induced this transformation with the patients in the clinic through the development of sense of coherence by development of character and purpose of life, which actually seems to be a very old strategy.

**TABLE 2**
**Study of 26 Patients Who's Ratings of Self-Esteem (Feelings about Themselves) were Changed with Therapy from Bad to Not Bad***

| | | Mean | N | Std. Deviation | Std. Error Mean |
|---|---|---|---|---|---|
| Physical health | Before | 2.5769 | 26 | 0.80861 | 0.15858 |
| | After | 2.1538 | 26 | 0.78446 | 0.15385 |
| Mental health | Before | 3.8000 | 26 | 0.81650 | 0.16330 |
| | After | 2.3600 | 26 | 0.99499 | 0.19900 |
| Self-esteem | Before | 4.0769 | 26 | 0.27175 | 0.05329 |
| | After | 2.2692 | 26 | 0.60383 | 0.11842 |
| Relation to friends | Before | 2.6923 | 26 | 1.04954 | 0.20583 |
| | After | 2.0385 | 26 | 0.82369 | 0.16154 |
| Relation to partner | Before | 4.6154 | 26 | 1.65111 | 0.32381 |
| | After | 3.0385 | 26 | 1.94896 | 0.38222 |
| Ability to love | Before | 3.8846 | 26 | 0.99305 | 0.19475 |
| | After | 2.3846 | 26 | 1.06120 | 0.20812 |
| Sexual ability | Before | 3.5000 | 26 | 1.02956 | 0.20191 |
| | After | 2.6154 | 26 | 1.09825 | 0.21538 |
| Social ability | Before | .3.3077 | 26 | 1.01071 | 0.19822 |
| | After | 2.2308 | 26 | 0.90808 | 0.17809 |
| Work ability | Before | 3.1538 | 26 | 1.00766 | 0.19762 |
| | After | 2.8462 | 26 | 1.22286 | 0.23982 |
| Quality of life | Before | 3.8462 | 26 | 0.73170 | 0.14350 |
| | After | 2.2308 | 26 | 0.86291 | 0.16923 |

* From 4 = bad or 5 = very bad, to 1 = very good, 2 = good, or 3 = neither good nor bad; paired samples statistics.

**TABLE 3**
**Study of 26 Patients Who's Ratings of Self-Esteem (Feelings about Themselves) were Changed with Therapy from Bad to Not Bad***

| | Paired Differences | | | | | t | df | Significance (Two–Tailed) |
|---|---|---|---|---|---|---|---|---|
| | Mean | Std. Deviation | Std. Error Mean | 95% CI of Difference | | | | |
| | | | | Lower | Upper | | | |
| Physical health | 0.4231 | 0.98684 | 0.19353 | 0.0245 | 0.8217 | 2.186 | 25 | 0.038 |
| Mental health | 1.4400 | 1.15758 | 0.23152 | 0.9622 | 1.9178 | 6.220 | 24 | 0.000 |
| Self-esteem | 1.8077 | .63367 | 0.12427 | 1.5517 | 2.0636 | 14.546 | 25 | 0.000 |
| Relation to friends | 0.6538 | 1.12933 | 0.22148 | 0.1977 | 1.1100 | 2.952 | 25 | 0.007 |
| Relation to partner | 1.5769 | 2.17574 | 0.42670 | 0.6981 | 2.4557 | 3.696 | 25 | 0.001 |
| Ability to love | 1.5000 | 1.20830 | 0.23697 | 1.0120 | 1.9880 | 6.330 | 25 | 0.000 |
| Sexual ability | 0.8846 | 0.95192 | 0.18669 | 0.5001 | 1.2691 | 4.738 | 25 | 0.000 |
| Social ability | 1.0769 | 1.09263 | 0.21428 | 0.6356 | 1.5182 | 5.026 | 25 | 0.000 |
| Work ability | 0.3077 | 1.43581 | 0.28158 | –0.2722 | 0.8876 | 1.093 | 25 | 0.285 |
| Quality of life | 1.6154 | 1.20256 | 0.23584 | 1.1297 | 2.1011 | 6.849 | 25 | 0.000 |

* From 4 = bad or 5 = very bad, to 1 = very good, 2 = good, or 3 = neither good nor bad; paired samples test.

**TABLE 4**
**Paired Samples Statistics**

| | | Mean | N | Std. Deviation | Std. Error Mean |
|---|---|---|---|---|---|
| Relations | Before | 3.7949 | 26 | 0.70602 | 0.13846 |
| | After | 2.4487 | 26 | 0.76561 | 0.15015 |
| Ability | Before | 3.4615 | 26 | 0.59453 | 0.11660 |
| | After | 2.5192 | 26 | 0.74473 | 0.14605 |
| QOL (QOL5) | Before | 3.6200 | 25 | 0.45775 | 0.09155 |
| | After | 2.3400 | 25 | 0.63915 | 0.12783 |
| Health-QOL-Ability (QOL10) | Before | 3.4800 | 25 | 0.44866 | 0.08973 |
| | After | 2.3878 | 25 | 0.64132 | 0.12826 |

**TABLE 5**
**Paired Samples Test**

| | Paired Differences | | | | | t | df | Significance (Two–Tailed) |
|---|---|---|---|---|---|---|---|---|
| | Mean | Std. Deviation | Std. Error Mean | 95% CI of Difference | | | | |
| | | | | Lower | Upper | | | |
| Relations | 1.3462 | 0.80267 | 0.15742 | 1.0219 | 1.6704 | 8.552 | 25 | 0.000 |
| Ability | 0.9423 | 0.82252 | 0.16131 | 0.6101 | 1.2745 | 5.842 | 25 | 0.000 |
| QOL (QOL5) | 1.2800 | 0.67309 | 0.13462 | 1.0022 | 1.5578 | 9.508 | 24 | 0.000 |
| Health-QOL-Ability (QOL10) | 1.0922 | 0.65752 | 0.13150 | 0.8208 | 1.3636 | 8.306 | 24 | 0.000 |

This healing of almost all aspects of life is often seen with clinical holistic medicine and is called (Antonovsky-) salutogenesis after the researcher who discovered this type of immediate, lasting, and all-inclusive healing of the patient’s existence. The most remarkable thing is that this seems to be the kind of healing that was induced by Hippocrates and his students 2,300 years ago on the island of Cos[33], where recovery of the human character was the primary tool for this. Physicians have been laughing for centuries about Hippocrates’ theory of “black and white bile” (the humeral medicine using the four elements), but it can still work wonders today to recover the human soul and character. It is possible because this is the door to the purpose of life, where we use our primary talents (often called our life-purpose or “mission of life”) in order to be of value to others[34] and give from our own gift to others.

Clinical holistic medicine, i.e., mindful psychodynamic short-term therapy complemented with bodywork, seems from this study to be the perfect tool for helping patients to recover their self-esteem. This can be done quickly, efficiently, cheaply, and without side effects. Most interesting, by successfully inducing existential healing (salutogenesis), almost all aspects of life were improved at the same time — physical and mental health, quality of life, ability to function in a number of important areas: with partner and friends, sexually, and socially. Most importantly from a philosophical point of view, the patient’s ability to love was recovered when the patient started to love him- or herself again. The strategy of improving self-esteem can be the key to a new life for chronic patients who present the triad of low quality of life, poor health (physical and/or mental), and poor ability to function. This combination is very difficult to help, not to say cure, by traditional biomedical or psychiatric treatment.

## CONCLUSIONS

A total of 43 patients entered the study with low or very low self-esteem, but after an average of 20 sessions, 26 persons (60.5%) were cured (95% CI: 44.41–75.02%). In clinical holistic medicine with patients with low self-esteem, NNT was thus calculated to NNT = 1.33–2.25. As we have treated more than 500 patients with no patient harmed, we estimate the NNH as >500.

The rate of recovery was comparable to the most successful interventions with psychological and psychiatric treatment, and as clinical holistic treatment seems to have almost no side effects, it seems to be the choice of treatment for the patients who are able to endure the emotional pain it provokes. On average, the patients received 20 treatments over a 14-month period at a cost of 1,600 EURO.

## ACKNOWLEDGMENTS

This study was supported by grants from IMK Almene Fond. The quality of life research was originally approved by the Copenhagen Scientific Ethical Committee under number (KF)V.100.2123/91 and later correspondence.

## References

1. Fromm, E. (2000) *The Art of Loving*. HarperCollins, New York.
2. Buber, M. (1970) *I and Thou*. Charles Scribner´s Sons, New York.
3. Jones, E. (1961) *The Life and Works of Sigmund Freud*. Basic Books, New York.
4. Jung, C.G. (1964) *Man and His Symbols*. Anchor Press, New York.
5. Antonovsky, A. (1985) *Health, Stress and Coping*. Jossey-Bass, London.
6. Antonovsky, A. (1987) *Unravelling the Mystery of Health. How People Manage Stress and Stay Well.* Jossey-Bass, San Francisco.
7. Ornish, D. (1999) *Love and Survival. The Scientific Basis for the Healing Power of Intimacy*. HarperCollins, Perennial, NY.
8. Chopra, D. (1990) *Quantum Healing. Exploring the Frontiers of Mind Body Medicine*. Bantam Books, New York.
9. Anderson, E.M. and Lambert, M.J. (1995) Short term dynamically oriented psychotherapy: a review and meta-analysis. *Clin. Psychol. Rev*. **15,** 503–514.
10. Crits-Cristoph, P. (1992) The efficacy of brief dynamic psychotherapy: a meta-analysis. *Am. J. Psychiatry* **149**, 151–158.
11. Svartberg, M. and Stiles, T.C. (1991) Comparative effects of short-term psychodynamic psychotherapy: a meta-analysis. *J. Consult. Clin. Psychol*. **59**, 704–714.
12. de Vibe, M. and Moum, T. (2006) [Training in mindfulness for patients with stress and chronic illness]. *Tidsskr. Nor. Laegeforen*. **126(15),** 1898–1902. [Norwegian]
13. de Vibe, M. (2003) [Mindfullness training--a method for self-regulation of health]. *Tidsskr. Nor. Laegeforen*. **123(21),** 3062–3063. [Norwegian]
14. Rosen, M. and Brenner, S. (2003) *Rosen Method Bodywork. Accessing the Unconscious Through Touch.* North Atlantic Books, Berkeley, CA.
15. Rothshild, B. (2000) *The Body Remembers*. W.W. Norton, New York.
16. van der Kolk, B.A. (1994) The body keeps the score: memory and the evolving psychobiology of post traumatic stress. *Harvard Rev. Psychiatry* **1**, 253–265.
17. Ventegodt, S., Flensborg-Madsen, T., Andersen, N.J., Nielsen, M., Mohammed, M., and Merrick, J. (2005) Global quality of life (QOL), health and ability are primarily determined by our consciousness. Research findings from Denmark 1991–2004. *Soc. Indicator Res.* **71**, 87–122.
18. Ventegodt, S., Kandel, I., and Merrick, J. (2005) *Principles of Holistic Medicine. Philosophy Behind Quality of Life*. Trafford, Victoria, BC.
19. Ventegodt, S., Kandel, I., and Merrick, J. (2005) *Principles of Holistic Medicine. Quality of Life and Health.* Hippocrates, New York.

20. Ventegodt, S., Kandel, I., and Merrick, J. (2005) *Principles of Holistic Medicine. Global Quality of Life. Theory, Research and Methodology.* Hippocrates, New York.
21. Ventegodt, S., Thegler, S., Andreasen, T., Struve, F., Enevoldsen, L., Bassaine, L., Torp, M., and Merrick, J. (2006) Clinical holistic medicine: psychodynamic short-time therapy complemented with bodywork. A clinical follow-up study of 109 patients. *TSW Holistic Health & Medicine* **1,** 256–274.
22. Ventegodt, S. (1995) *Quality of Life. To Seize the Meaning of Life and Become Well Again.* [*Livskvalitet – at erobre livets mening og blive rask igen.*] Forskningscentrets Forlag, Copenhagen. [Danish]
23. Ventegodt, S. (2003) *Consciousness-Based Medicine.* [*Bevidsthedsmedicin – set gennem lægejournalen.*] Forskningscentrets Forlag, Copenhagen. [Danish]
24. Lindholt, J.S., Ventegodt, S., and Henneberg, E.W. (2002) Development and validation of QoL5 clinical databases. A short, global and generic questionnaire based on an integrated theory of the quality of life. *Eur. J. Surg.* **168,** 103–107.
25. Diem, K., Ed. *Documenta Geigy. Scientific Tables.* Several editions, 1962 and later. J.R. Geigy S.A., Basel.
26. Goodson, P., Buhi, E.R., and Dunsmore, S.C. (2006) Self-esteem and adolescent sexual behaviors, attitudes, and intentions: a systematic review. *J. Adolesc. Health* **38(3),** 310–319.
27. Kermode, S. and MacLean, D. (2001) A study of the relationship between quality of life, health and self-esteem. School of Nursing and Health Care Practices, Southern Cross University, Lismore, Australia. *Aust. J. Adv. Nurs.* **19(2),** 33–40.
28. Shimizu, M. and Pelham, B.W. (2004) The unconscious cost of good fortune: implicit and explicit self-esteem, positive life events, and health. *Health Psychol.* **23(1),** 101–105.
29. Willoughby, C., Polatajko, H., Currado, C., Harris, K., and King, G. (2000) Measuring the self-esteem of adolescents with mental health problems: theory meets practice. *Can. J. Occup. Ther.* **67(4),** 230–238.
30. Mann, M., Hosman, C.M., Schaalma, H.P., and de Vries, N.K. (2004) Self-esteem in a broad-spectrum approach for mental health promotion. *Health Educ. Res.* **19(4),** 357–372.
31. MacInnes, D.L. (2006) Self-esteem and self-acceptance: an examination into their relationship and their effect on psychological health. *J. Psychiatr. Ment. Health Nurs.* **13(5),** 483–489.
32. Berge, M. and Ranney, M. (2005) Self-esteem and stigma among persons with schizophrenia: implications for mental health. *Care Manage. J.* **6(3),** 139–144.
33. Jones, W.H.S. (1923–1931) *Hippocrates.* Vol. I–IV. William Heinemann, London.
34. Ventegodt, S. (2003) The life mission theory: a theory for a consciousness-based medicine. *Int. J. Adolesc. Med. Health* **15(1),** 89–91.
